# Clinical and Environmental Surveillance for the Prevention of Legionellosis

**DOI:** 10.3390/microorganisms12050939

**Published:** 2024-05-06

**Authors:** Maria Anna Coniglio, Mohamed H. Yassin

**Affiliations:** 1 Regional Reference Laboratory of Clinical and Environmental Surveillance of Legionellosis, Department of Medical and Surgical Sciences and Advanced Technologies “G.F. Ingrassia”, University of Catania, Via Sofia 87, 95123 Catania, Italy; 2 Azienda Ospedaliero Universitaria Policlinico “G. Rodolico-San Marco”, Via S. Sofia 78, 95123 Catania, Italy; 3 Infectious Diseases and Infection Prevention Department, University of Pittsburgh, School of Medicine and Public Health Pittsburgh, Pittsburgh, PA 15213, USA; yassinm@upmc.edu

*Legionella* is a Gram-negative bacterium whose natural hosts are aquatic protozoa, in which the microorganism replicates and is protected from adverse environmental conditions. From its natural reservoirs, the bacterium can colonize water systems in different types of buildings (e.g., hospitals, hotels, or private homes), thermal baths, spas, and dental units. Subsequently, *Legionella* can be transmitted by inhaling infected aerosols from showerheads, certain medical or hydrotherapy equipment, cooling towers, as well as decorative fountains [1]. 

Among the different species, *Legionella pneumophila* is most frequently associated with human disease, causing a serious type of pneumonia, the so-called Legionnaire’s disease, or a mild flu-like illness called Pontiac fever. Nonetheless, other species, including *L. bozemanae*, *L. dumoffii*, and *L. longbeachae,* can also cause human infections [2].

The isolation and identification of *Legionella* from the environment is crucial for managing environmental and clinical prevention, as well as for epidemiological purposes and outbreak investigations. Epidemiological data combined with microbiological and clinical information can contribute to identifying the source of infection and implementing control measures. Thus, it is necessary to promote environmental and clinical surveillance programs, improve diagnostic techniques, and set up preventive measures [3]. 

Undoubtedly, the epidemiology of legionellosis has recently been affected by the prevention measures adopted against COVID-19. National-level data on hospitalization trends, clinical surveillance, and environmental monitoring for *Legionella* are crucial in understanding the temporal and geographical trends of Legionnaires’ disease. During the pandemic period, governments worldwide took various measures to restrict travel in order to limit the spread of coronavirus. Subsequently, in UE/EEA countries, the number of reported cases of travel-associated legionellosis decreased by 67% in 2020 compared with 2019 [4]. On the other hand, the pandemic period significantly increased the total number of diagnoses of hospital-acquired Legionnaire’s disease. In particular, a high prevalence of legionellosis among SARS-CoV-2 patients was found in hospitals that adopted differential diagnostic protocols for cases of pneumonia, which included both the tests for the search for SARS-CoV-2 and urine antigens of *Legionella* [5]. 

Environmental routine testing for *Legionella* can also help safeguard the health of building occupants and visitors to any facility, as well as prevent the risk of legionellosis associated with the work environment. Furthermore, regular environmental monitoring can prevent potential outbreaks of Legionnaires’ disease. The development of new approaches for implementing water management programs, together with the application of new technologies to study *Legionella*, will undoubtedly provide new insights with broad implications at clinical and environmental levels [6]. For this purpose, the new EU Drinking Water Directive (2020/2184) [7] recommends a risk-based approach for the proper management of *Legionella*. 

A multidisciplinary approach for an accurate risk evaluation for the prevention of legionellosis in hospital and community settings could help increase monitoring sensitivity. Unfortunately, the standard ISO 11731 [8] method fails to detect viable but non-culturable (VBNC) Legionella [9], which is both metabolically active and infective in the VBNC state [10,11]. Thus, analytical strategies that combine traditional culture methods and innovative characterization techniques should be considered when developing a water management program. Furthermore, mathematical predictive analyses and statistical models could help improve environmental risk assessment and management, as well as forecast future outcomes and scenarios based on environmental surveillance. In particular, bioinformatics, in association with bacterial genome sequencing technologies, are becoming widely used techniques in clinical diagnostics and public health. Sequencing technologies enable the high-resolution characterization of *Legionella* not only in terms of molecular epidemiology but also in terms of virulence, and the association with bioinformatics analysis can provide a reliable molecular surveillance tool [12]. This could also be useful in preventing large-scale outbreaks of Legionnaires’ disease, as well as in performing comparative genetic analyses of clinical and environmental isolates in cases of outbreak. 

Taking into consideration what has been mentioned above, this Special Issue, Clinical and Environmental Surveillance for the Prevention of Legionellosis, has contributed an overview of the most recent advances not only in the field of environmental surveillance and clinical diagnosis but also in the management of healthcare and community-acquired legionellosis. In particular, under the umbrella of this research topic, 10 publications have highlighted the current extent of *Legionella* research and the future direction in the field of environmental surveillance, clinical diagnosis, and applications of innovative monitoring methods with regard to sequencing technologies. 
